# Fly-Ash Pollution Modulates Growth, Biochemical Attributes, Antioxidant Activity and Gene Expression in *Pithecellobium Dulce* (Roxb) Benth

**DOI:** 10.3390/plants8120528

**Published:** 2019-11-20

**Authors:** Sami Ullah Qadir, Vaseem Raja, Weqar Ahmad Siddiqui, Mahmooduzzafar, Elsayed F. Abd_Allah, Abeer Hashem, Pravej Alam, Parvaiz Ahmad

**Affiliations:** 1 Analytical Research Laboratory Department of Applied Sciences and Humanities, Faculty of Engineering and Technology Jamia Millia Islamia, New Delhi 110025, India; samiullahqadir@gmail.com (S.U.Q.); wsiddiqui@jmi.ac.in (W.A.S.); 2 Department of Botany, Jamia Hamdard University, New Delhi 110062, India; wrajamp2009@gmail.com (V.R.); mahmooduzzafar01@gmail.com (M.); 3 Department of Botany, Govt. College for Women, Baramulla 193101, Jammu and Kashmir, India; 4 Department of Plant Production, Faculty of Food & Agricultural Sciences, King Saud University, Riyadh 11451, Saudi Arabia; elsayed_22@yahoo.com; 5 Botany and Microbiology Department, College of Science, King Saudi University, Riyadh 11451, Saudi Arabia; abeer.hashem@gmail.com; 6 Mycology and Plant Disease Survey Department, Plant Pathology Research Institute, ARC, Giza 12511, Egypt; 7 Department of Biology, College of Science and Humanities, Prince Sattam bin Abdulaziz University, Alkharj 11942, Saudi Arabia; pravejalam93@gmail.com; 8 Department of Botany, S.P. College, Srinagar 190001, Jammu and Kashmir, India

**Keywords:** coal combustion, fly ash, oxidative stress, antioxidant enzymes, air pollution tolerance index

## Abstract

This study investigates the effect of fly ash (FA) on the *Pithecellobium dulce* (Roxb) Benth. trees growing at three different locations. FA stress caused significant changes in different leaf attributes like sugar, protein contents, photosynthetic pigments, nitrate content and nitrate reductase activity in foliar tissues of plants growing at a highly contaminated site, as compared to a low-pollution site. Lower rates of stomatal conductance (SC) were observed in *P. dulce* leaves under fly ash stress conditions that drastically reduced net photosynthetic rate (P_N_); however, intercellular carbon dioxide concentration and stomatal index (SI) showed an increase under the same stress conditions. On the other hand, significant increase was also observed in the proline, sulphur and nitrogen contents. A significant increase in oxidative stress and, consequently, in antioxidant enzymes such as ascorbate peroxidase (APX), catalase (CAT), peroxidase (POD), and superoxidase dismutase (SOD) and Air pollution tolerance index were discovered at three different sites. The transcriptional expression of antioxidant and stress responsive genes was higher at HPS as compared to two other two sites of the study. Taken together the results demonstrated that the *P. dulce* is best suited as a fly ash stress tolerant plant species with the potential to provide an alternative for the reclamation of fly ash affected soils.

## 1. Introduction

Fly ash or flue ash (FA) is the coal combustion residue composed of fine particles of burned fuel, produced in bulk amounts from thermal power plants. Globally, FA generation from the thermal power plants is expected to increase to the amount of approximately 300–600 million tons per annum by the year 2020 [1], which may cover up to 3235 km^2^ of land area for its disposal [1], while FA production in India is likely to be 300–400 million tons per annum by this period. FA generated from thermal power plants is disposed of either by wet or by dry methods. In case of dry disposal methods, FA is directly discarded in fly ash basins and landfills, while in the case of wet disposal methods, FA is often eroded out with water and piped as slurry into settling ponds, lagoons or artificial dams. Over the time, water from these settling ponds is permitted to channel away, and the ash left behind is often characterized as pond ash. Both of the disposal methods effectually encourage FA dumping of in landfills on open land. As a result, FA deposits exert harmful effects on the neighboring aquatic, terrestrial and aerial bionetworks. Fly ash disposal as the landfill is under compression from environmental disquiets and progressively rigorous environmental regulations are steadily ballooning the disposal costs [2]. In this direction, negligence toward FA dumps may lead to contamination of the environment to such an extent that may be alarming for both human health and day to day living [3,4]. In addition to toxic heavy metals such as polycyclic aromatic hydrocarbons (PAHs), Cr, Cd, As, Hg, Pb, etc, are the chief pollutants of fly ash that lead to the contamination of soil, air, and water resources in the surrounding areas of coal-based thermal power plants. More often these pollutants become the serious threat for the adjacent vegetation [4,5,6,7,8]. Fly ash lagoons pose serious threats to human life by virtue of their ability to cause various skin, cardiac, genetic and respiratory problems and cancer [9].

All of the ecological problems arising due to disposal and management of FA can be diminished by the plantation of ash dumpsites. For the successful reinstatement of FA lagoons, analysis of adaptability and rejuvenation potential of plants in the FA ecosystem is desirable. Factually the hostile substrate and local environments are the strategic factors that hamper natural plant colonization on FA dumps [10]. Appropriate phytoremediating plant species should be planted to increase the potential of phyto-colonization on these FA dumpsites [11,12,13]. Among the key factors, that hamper plant growth and survival on FA dumping sites are deficiency of essential plant nutrients, mostly high boron content, available phosphorus (0.05–0.2%) and nitrogen (<0.05) [14]. Plantation checks erosion of FA and controls the leaching of noxious elements, either through binding of ash particles by their roots or by plant uptake [15]. Rigorous and unremitting deposition of contaminants on leaves of plants can cause several physiological disorders, decline in plant cover and, above all can lead to the vanishing of fragile species from the pollution-exposed sites.

Higher levels of these pollutants become phytotoxic and are often accompanied with reactive oxygen species (ROS) production. Pollutant-induced oxidative damage with overproduction of ROS in the form of superoxide radicals (O_2_^−^), hydroxyl radicals (OH^−^), and hydrogen peroxide (H_2_O_2_), which can rapidly interact with proteins, lipids, and DNA, may result in cell death [16,17,18]. Regardless of their damaging activity, ROS are deliberated to act as the second messenger actively involved in several signaling processes [19]. Plants respond to environmental insults by changing their biochemical, physiological and transcriptomic levels to the promising level. By the use of this composite machinery, plants may avert the damage and safeguard survival under harsh conditions [20], but often at the cost of condensed growth and productivity [21].

Thus, biostabilization is considered to be an operative resolution against environmental pollution of FA dumpsites [22]. Numerous investigators have evaluated the FA-grown plant species for their bioaccumulation potential for heavy metal remediation of FA dumps [9,23]. There are a few reports in regard to natural vegetation on FA deposits across the world, but a lesser amount of information is available about the phytodiversity of these FA deposits [24]. Given the above, this study attempts to recognize the potential impact of FA stress on some physiological, morphological and biochemical features of *P. dulce*, a naturally growing tree species at the FA dumpsite of Badarpur Thermal Power Plant (BTPP) in Delhi, India.

## 2. Materials and Methods

### 2.1. Study Area and its Climate

Delhi, the capital of India, is located in the subtropical belt between 28°12′–28°53′ North and 76°50′–77°23′ East, roughly 216m above the sea level, with a topographical area of about 1483 sq. km (53% rural, 47% urban). The climate of Delhi is a monsoon-influenced, humid subtropical type with scorching, moist summers and temperatures ranging regularly from 20 °C to 41 °C. Monsoons in Delhi commence from the month of July and continue to September with a medium to heavy precipitation. The winters in Delhi are relatively cold with the temperature falling to as low as 7 °C due to a cold wave from the Himalayan province.

### 2.2. Study Site Selection

Three experimental sites (I, II, and III) for the present study were selected at about 0.5, 5, and 17 km, respectively, from the Jamia Millia Islamia (JMI), a central university, to BTPP, a fly ash dumping site, downstream of the wind direction, because of the frequent flow of winds in this particular direction predominantly in the monsoon months. Being an academic area JMI, site (I) was selected as the control site as it was free from any pollution-causing sources. Compared to the other two sites (II and III), data on air quality revealed significantly lower concentrations of pollutants. Soil quality observed at all the three study sites was found to be sandy loam with coarse texture. The pH values of soil observed during the present study ranged from 6.69 to 7.16 at three different experimental sites. The study area had noticeably wet and dry periods due to the seasonality of air pollution differences. For the present study, winter season (Oct–Feb) was selected because concentrations of pollutants tend to be at maximum during this particular period [25].

### 2.3. Sampling of Plant Material

At each sampling location, five trees of *P. dulce* (family Fabaceae) were marked and the age of all the trees was calculated on the measured tree parameters, the diameter at breast height (dbh), and the total tree height as in [26]. From each tree, five fully expanded healthy leaves were collected from the two upper twigs fully exposed to sunlight. Thus, 75 leaves collected from three different locations were used for this study.

### 2.4. Measurement of pH and Suspended Particulate Matter (SPM)

The leaf extract pH was measured by following the method of [1]. Slurry of leaf material was prepared by homogenizing 0.5 g of leaf material in 50 mL of deionized water. The homogenate was centrifuged for 5 min at 10,000× *g*, and the pH was measured from the supernatant with the help of pH meter (Mettler-toledo S-20). While for the pH measurement of FA and soil a known amount of FA and soil was dissolved the in double distilled water, the suspension was agitated at regular intervals and pH was recorded [25]. SPM was determined through the use of a High Volume Air Sampler (Envirotech, Redwoodcity, CA, USA).

### 2.5. Epidermal Studies

Epidermal studies in the leaves of *P. dulce* plants were carried out according to Ghouse and Yunus [27]. For microscopic analysis leaves were cut in 2 cm × 2, rectangular pieces heated with 60% nitric acid until peels got separated, and thoroughly washed with double distilled water, using safranin as a coloring agent, the peels were mounted on glass slides using Canada balsam. The procedure of Salisbury [28] was used for the calculation of Stomatal index (S.I.). The peels were also observed under Scanning Electron Microscope, using the procedure described by [29].

### 2.6. Photosynthesis and Photosynthetic Pigments

Leaf area measurements were carried out according to Qadir, et al. [14] using Leaf Area Meter (SYSTRONICS, 211 India). Photosynthetic parameters like carbon dioxide assimilation rate, stomatal conductance and net photosynthetic rate (P*n*), were measured in the morning between 7:00 and 9:30 using IRGA (LI-COR, Lincoln, NE, USA). Photosynthetic pigments were carried out through DMSO extraction method of Hiscox and Israelstam [30]. Calculations of the values were carried out according to the formulae of Duxbury and Yentsch [31] and Maclachlan and Zalik [32].

### 2.7. Nitrogen Assimilation Related Parameters

Nitrate reductase activity (NR) in the leaf samples was performed according to Klepper et al. [33]. Nitrate measurements were performed by Grover, et al. [34]. Fresh leaves were collected from the study sites washed with deionized water and treated with a freshly prepared solution of CuSO_4_–ZnSO_4_, charcoal, 0.1N NaOH, and hydrazine sulphate. Reaction was stopped by using chilled acetone, for color development NEDD and Sulphanilamide was added. Spectrophotomatric analysis of the chromophore at 540 nm was performed according to Evans and Nason [35]. A condensed amount of nitrogen in leaf samples was estimated by Lindner [36] method.

### 2.8. Sugar, Sulphur, Proline, and Protein Contents

Total soluble sugar content was projected according to Dey [37] method, using sulphuric acid, 5% phenolethyl alcohol, and absorbance of the solution was recorded at 485nm after cooling to room temperature. Total amount of sulphur in leaf materials was estimated as per Chesnin and Yien [38]. The amount of free proline was evaluated using the ninhydrin method [39]. Total soluble protein measurements in fresh leaves were performed following the Bradford [40] method, 100 µL crude protein extract was thoroughly mixed with 1 mL of freshly prepared Bradford solution and the absorbance was recorded at 595 nm, using bovine serum albumin as standard.

### 2.9. Air Pollution Tolerance Index (APTI)

For assessing APTI, four parameters, viz. Ascorbic Acid, leaf extract pH, RWC, and total chlorophyll content from the plant material were calculated. AA content was measured according to Keller and Schwager [41] and RWC was determined as per Qadir and Siddiqui [42] by using the mathematical expression (1). Mathematical expression (2) was used to calculate APTI as proposed by Singh, et al. [43].

(1) $$RWC=\left[\frac{FW-DW}{TW-DW}\right]\times 100$$

where, TW = Turgid weight, DW = Dry weight FW = Fresh weight

(2) $$\mathrm{APTI}=\frac{\left[AA\;\left(T+P\right)+R\right]}{10}$$

where, R=Relative water content of leaf (%), P = pH of the leaf extract, AA=Ascorbic acid content (mg/gm.), extract and T = Total chlorophyll (mg/gm.), RWC = Relative water content.

### 2.10. Estimation of Malondialdehyde (MDA) and Antioxidant Enzyme Activities

MDA content from the leaf samples was measured as described by Heath and Packer [44]. For antioxidant assays, fresh leaves were selected randomly and mixed. 0.5 g freshly collected leaf material was macerated in 7 mL of 50 mM phosphate buffer (pH 7.8) in pre-chilled pestle and mortar containing 1% polyvinyl pyrrolidine (PVP) and 1 mM EDTA at 4 °C and centrifuged at 12,000× *g* for 40 min at 4 °C and the resulting upper layer was carefully aspirated and collected in pre-cooled fresh eppendorf tubes to determine different enzyme activities.

### 2.11. Ascorbate Peroxidase (APX EC1.11.1.11)

APX activity was assessed as per Nakano and Asada [45] method. Reaction mixture consisted of 3 mL enzyme extract (100 μL), 0.3 mM ascorbic acid, 100 mM phosphate (pH 7.0), 0.06 mM H_2_O_2_ and 0.1 mM EDTANa_2_. The variation in absorption was noted at 290nm after the addition of H_2_O_2_ for 2 min at every 30 s interval.

### 2.12. Catalase (CAT, EC1.11.1.6)

CAT activity was determined according to the method of Aebi [46] by observing H_2_O_2_ decomposition for 2 min at 240 nm using spectrophotometer (UV-2600 SHIMADZU, Kyoto, Japan). Reaction mixture (3 mL) consisted of 100 μL enzyme extract, 50 mM phosphate buffer (pH 7.0), 2 mM EDTA Na_2_ and 10 mM H_2_O_2_.

### 2.13. Superoxide Dismutase (SOD, EC1.15.1.1)

SOD enzyme activity was performed according to the method of Zhang, et al. [47]. Reaction mixture (3 mL) was prepared by dissolving 100 μL of enzyme, 1.5 mL of 100 mM phosphate buffer (pH 7.8), 75 μM NBT, 0.2 mL of 10 mM methionine, 0.1 mL of 50 μM riboflavin and 0.1 mM EDTA. The tubes containing the reaction mixture were placed under florescent light for about 20 min before the absorbance was measured at 560 nm.

### 2.14. Peroxidase (POD, EC1.11.1.7)

POD activity was performed by following Zhou and Leul [48] method. Reaction mixture (3 mL) consisted of enzyme extract (100 μL), 1.5 mL of 100 mM phosphate buffer (pH 7.0), 0.4% H_2_O_2_ and 1% guaiacol. The absorbance was determined at 470 nm. The increase in absorbance caused due to guaiacol oxidation was estimated at every 1 min interval for 4 min.

### 2.15. Expression Analysis of Antioxidant and Stress Responsive Genes

#### 2.15.1. Primer Design

Primers corresponding to antioxidant and stress related genes were designed on the basis of gene sequences. Primer sequences and target genes are listed here, Ascorbate Peroxidase (APX): Forward 5′ TTC GAT GGG TTG TGA TTT GA 3′, Reverse 5′ CGT TGC GTT AGA CTT GTT TT 3′; Superoxide Dismutase (SOD): Forward 5′ ACT ATC TTC TTC ACC CAG GA 3′, Reverse 5′ GAG TTT GGT CCA GTA AGA GG 3′; Peroxidase (POD): Forward 5′ CCG AAG CAT GAT TGG AGC AC 3′, Reverse 5′ AGC GCA GCA TCC GAA TCT AT 3′; Catalase (CAT): Forward 5′ AAC CAT GAG GGA TAT TCG TG 3′, Reverse 5′ TGG ATG TTA GTT TTC GGG TT 3′; Late Embyrogenisis abundant Proteins (LEA): Forward 5′ GGA AGC ATG AAG CCGGA 3′, Reverse 5′ AGT CGA GGT CCC AAT CCG TA 3′; Dehydration Responsive Element binding factor (DREB): Forward 5′ TGG CGT TAG GGT TTT CCG AT 3′, Reverse 5′ GCG GGT GCT TTT CGA GTT TT 3′; Tubulin (TB): Forward 5′ GAT AAC TGT ACT GGA CTG CAAGG 3′, Reverse 5′ GGA TGG CTT CGT TAT CCA AGAG 3′. Software Primer 3 was used for primer selection and were prepared commercially (Integrated DNA Technologies IDT, Coral ville, IA, USA). Tubulin gene which served as an internal control was also amplified along with other genes.

#### 2.15.2. RNA Extraction and Semi-Quantitative PCR Analysis

RNA from fresh leaves was extracted by using Trizol reagent (Thermo Scientific, Weltham, MA, USA). The Purification was carried out by passing the RNA through on-column DNaseI and RNAeasy spin column. Eluted RNA was quantified spectrophotometrically and the quality was analyzed through agarose gel electrophoresis. cDNA synthesis was performed by using RevertAid cDNA synthesis kit (Thermo Fisher Scientific) according to the supplier’s instructions. PCR reaction was carried out in a final reaction mixture of 50 μL containing 1 μL of cDNA, 0.1 mM dNTPs, 1X PCR buffer, 0.5 unit of Taq DNA polymerase (Sigma-Aldrich), 1μM primer (each reverse and forward) and nuclease free water. Amplification was carried out with thermal cycler (Veriti-96 Applied Biosystems) using following condition 2 min at 95 °C and then 35 cycles for 32 s each at 95 °C, 35 s at 54–57 (annealing temprature optimized for each gene), and 40 s for 72 °C followed by a final step of 10 min at 72 °C. PCR amplification products were subjected to gel electrophoresis, stained with ETBR, and visualized under UV light, gels were scanned in the Gel Documentation System (Kodak, Rochester, NY, USA).

#### 2.15.3. Quantitative Real-Time PCR

Quantitative real-time qPCR was carried out with the help of ABI 7300 RT-PCR system (Applied Biosystems) with PowerUP^TM^ SYBR Green Master Mix. Amplification of antioxidant and stress related genes (*APX*, *SOD*, *POD*, *CAT*, *LEA,* and *DREB*) was achieved as per manufacturer’s protocol. PCR reaction of about of 20 μL consisted 12.5 μL of 2X PowerUP^TM^ SYBR Green Master Mix, 10 ng of cDNA template, 300 nM primer (reverse and forward), and nuclease free water. The following conditions were used for thermocycling: 2 min at 50 °C, 10 min at 95 °C and 40 cycles alternating between 15 s at 95 °C and 1 min at 60 °C to verify primer specificity melting curve analysis (65–95 °C) was routinely performed after 40 cycles. A single amplified product for all genes was observed from the melting curves. For each sample three analytical replicates were used for the calculation of expression levels of the individual genes along with tubulin as an internal control and recorded as *Ct* at the default threshold (0.2). *Ct* was transformed to quantities relative to the sample and *C*t values of the used target genes were normalized using the *C*t values of tubulin, mRNA levels of the genes were normalized with that of the tubulin [49]. 2^−ΔΔ*C*t^ method [50] was used for the calculation of relative expression levels of the genes in respect to that of control.

### 2.16. Statistical Analysis

Experiments were conducted in five replications. Data was analyzed using IBM SPSS statistics 16.0 software and were articulated as mean ± SD. ANOVA with Duncan’s multiple range test as a post-hoc test (*P* < 0.05) was applied to calculate significant differences between three sites.

## 3. Results and Discussion

Suspended particulate matter (SPM) was monitored weekly in various localities of Delhi, data indicating that the JMI site (I), i.e., low pollution site (LPS) was least charged with SPM as the lowest mean value recorded for the SPM was (209.08 ± 19.68 µg m^−3^) followed by site (II) of medium pollution (MPS) with average SPM concentration of (221.34 ± 33.33 µg m^−3^), while the dumping site of BTPP site (III) was the high pollution site (HPS), showing very high SPM levels of (254.98 ± 62.50 µg m^−3^). Generally, the load of pollution depends on the levels of emission sources, and micrometeorological factors [51,52]. During the present study it was observed that site III was most polluted possibly due to significantly higher FA concentration because of open dumping of FA, together with other pollutants released into the atmosphere. Contrary to this, JMI site (I), was protected by high boundary walls with good tree density, restricted vehicular traffic and no industrial activity in its vicinity.

Plants exposed to pollution experience numerous morphological, physiological, biochemical and ultrastructural changes upon prolonged exposure [51,53]. Table 1 reflects the values of leaf traits such as leaf width, length and SLA, of *P. dulce* growing at three different sites. With increasing pollution load all the leaf attributes showed a decreased trend. A significant variation was noticed among these plant characteristics (leaf width, *F* = 503.60, *P* < 0.001; leaf length, *F* = 128.77, *P* < 0.001; SLA, *F* = 246.83, *P* < 0.001), showing a variation of 51%, 45% and 66% between LPS and HPS, respectively.

Contrary to control site, dust load is considered to be an important factor responsible for the decline in leaf performance at the contaminated site [25]. They reported a reduction in the SLA in plant leaves growing at a highly industrial polluted area of Chunar, Mirzapur of India in comparison to that of low pollution area of the Banaras Hindu university campus. Similar results were reported in *Azadirachta indica* [14] under FA stress. Reductions in the leaf area might be attributed to decreased leaf production, and/or higher rates of senescence caused by the impact of cement dust pollution stress [54,55,56] on the photosynthesis capacity and cell elongation mechanism [57].

**Figure 1:**
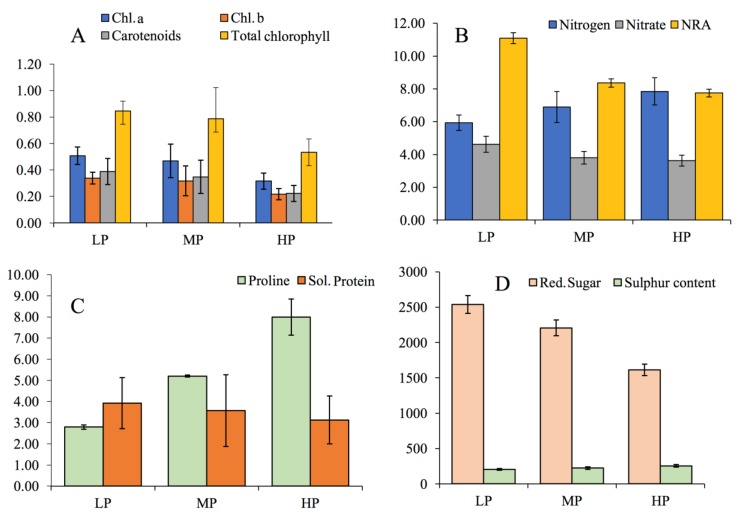
Variation in the biochemical parameters of *P. dulce* at three different sites (**A**) Chl. *A,* Chl. *b*, carotenoids, total chlorophyll (mg g^−1^ fw) (**B**) Nitrate and NRA (mmolg^−1^ fw) (**C**) protein (mg g^-1^ fw) and proline (µg g^−1^ fw) (**D**) Sulphur (mg g^−1^ dw) and reducing sugar (mg g^−1^ fw). Data represents mean ± SD (*n* = 5).

Varying degrees of foliar necrosis and chlorosis observed at HPS indicate degradation of photosynthetic pigments in the leaf tissue. The site-specific variation in biochemical properties of *P. dulce* are presented in Figure 1a–d. The amount of Chlorophyll “*a*” decreased with an increase in FA stress. It was 0.51 mg g^−1^ at LPS which declined to 0.47 mg g^−1^ (7.84%) at MPS and 0.32 mg g^−1^ (37.25%) at HPS. In relation to the intensity of FA pollution considerable differences were also observed in Chlorophyll “*b*”, total chlorophyll and carotenoids, in the foliar tissue of plants growing at different sites. Chlorophyll *b* content was 0.33 mg g^−1^ at LPS, 0.32 mg g^−1^ at MPS and 0.22 mg g^−1^ at HPS, displaying a maximum decline of 35.30% at HPS, in comparison to LPS. Total chlorophyll and carotenoid contents also shows a declining trend from LPS to HPS, the maximum reduction being 37.64%, and 43.58%, respectively, as shown in Figure 1a. From ANOVA, significant variation in the plant pigments at three different sites was noticed (Chlorophyll *a, F* = 32.00, *P* < 0.01; Chlorophyll *b, F =* 19.11, *P* < 0.01; Carotenoids, *F* = 19.07, *P* < 0.01; total chlorophyll, *F* = 29.14, *P* < 0.01).

Chlorophyll measurement under stressful environments is considered to be an imperative tool to assess the effects on plants because of its direct involvement in several metabolic processes. Any reduction in chlorophyll content has direct bearance on growth, productivity and tolerance [52,58,59]. Our study reports a significant reduction (*P* < 0.01) in plant pigments which may be associated with chloroplast damage by FA pollution stress. Reduction in pigment concentration of foliage surfaces may be attributed to the shading effect of FA particles at the polluted site than at the control [59]. FA particles might clog stomata, which leads to intensification in leaf temperature and interference with the gaseous exchange that may subsequently impede chlorophyll synthesis [25]. It is well documented that plants thriving in polluted environments often display alarming levels of photosynthetic pigments like chlorophyll “*a”,* chlorophyll *“b”* and total chlorophyll [60]. Pollution-induced photosynthetic pigment degradation was also documented in some recent studies [53,59,61,62]. Leaf surface crust formation of an alkaline nature is also deliberated to be one of the chief factors that contributed to the reduction in photosynthetic capacity under polluted environments [63]. It is assumed that at a site with maximum pollution load, chlorophyll “*a*” is often degraded to pheophytin and the formation of Chlorophyllide “*b*”occurs by the removal of phytol group from chlorophyll “*b*” [62] which is no longer capable to harvest solar energy for photosynthesis thereby resulting in a decrease in the chlorophyll pigments [61].

Carotenoids are an assembly of fat-soluble natural pigments associated with the photosynthetic process in photosynthetic bacteria, algae and plants. Several workers have also reported the loss of carotenoid pigments due to the action of pollutants [14,53,64]. Carotenoids prevent photo-oxidation of chlorophyll by acting as an antioxidant, but this normal defensive process of carotenoids is vulnerable to environmental stress and often results in cellular devastation, including pigment dilapidation [64].

With the increase in pollution stress from LPS to HPS nitrogen content in leaves showed an increasing trend. Nitrogen content at LPS was 5.94 mmolg^−1^ which increased to 6.90 mmolg^−1^ (16.16%) at MPS and 7.85 mmolg^−1^ (32.15%) at HPS. Maximum reduction of 21.42% and 30.14% between LPS and HPS were observed in the case of nitrate content and nitrate reductase activity respectively (Figure 1b). Significant variations among these parameters were observed at all study sites (nitrogen *F* = 38.22, *P* < 0.01; nitrate *F* = 41.98, *P* < 0.01; NRA, *F* = 4.51, *P* < 0.05). We found a significant positive relationship between FA stress and nitrogen content of plant leaves (*r* = 0.414, *P* < 0.01). Values corresponding to correlation coefficient between sites and various morpho-physiological attributes, antioxidant enzymes and biochemical parameters showed a significant relationship, as given in Table 2. Several researchers have reported an upsurge in the foliar nitrogen content in plants under pollution-stressed environments [14,25,53]. Nitrate reductase is metalloflavo protein inducible enzyme that plays a key role in the assimilatory reduction of nitrate to ammonia using NADH as an electron donor. NR activity is severely affected by metals, drought and salinity stress. Since FA has a low water-retention capacity coupled with high pH, therefore a decline in NRA of plants growing on raw fly ash might be due to low availability of substrate, salinity stress due to presence of salts, and an uptake of toxic metals from FA, which may exchange active metal sites of enzymes or generate active oxygen species that cause oxidation and cross-linking of SH groups [65]. The present study shows a strong negative relationship between sites and nitrate concentration of *P. dulce* (*r* = −0.69, *P* < 0.01). Pollution stress may negatively or positively affect foliar nitrate content. Fast-growing species often assimilate higher quantities of nitrates besides increased NR activities to put up an increased available soil nitrate. Similar results have been previously observed in *Azadirachta indica* [14,53].

Similar to pigment contents, a reduction in leaf protein content was also observed at polluted sites. During the present study, a maximum reduction of 20.36% in the case of leaf protein content was observed at HPS in comparison to LPS (*F* = 2.14, *P* > 0.05). In the case of proline, significant increase (*F* = 678.92, *P* < 0.01) was noticed from LPS to HPS, showing an increment of 185%, as shown in Figure 1c. A significant negative relationship existed between sites and soluble protein (*r*= −0.24, *P* < 0.05) (Table 3). Reduction in protein content in response to FA stress might be attributed to the breakdown of existing protein to amino acids, higher rates of protein denaturation and/ or reduced de novo protein synthesis [53,55]. From the collected data a positive correlation (*r* = 0.974; *P* < 0.001) exists between FA stress and proline accumulation (Table 3). Proline accumulation could be the result of de nevo synthesis, lower utilization, and decreased degradation hydrolysis of proteins. As multifunctional amino acid proline seems to have diverse roles under stressful conditions such as stabilization of subcellular structures, membranes and proteins, besides acting as an outstanding osmolyte, also plays the role of a metal chelator and acts as an antioxidant molecule [66]. Several researchers have reported that elevated proline levels in plants under stressful environmental conditions could impart stress tolerance by sustaining osmotic stability or cell turgor and protect cellular functions by scavenging ROS, thus checking oxidative burst in plants. An increase in proline levels under stressful environments was also reported [14,53,66].

Foliar sugar content decreased significantly in the leaves of *P. dulce* under fly ash stress (*F* = 4.04, *P* < 0.05). Foliar sugar content perceived at LPS was 2537.76 µg g^−1^, 2206.82 µg g^−1^ at MPS and 1613.62 µg g^−1^ at HPS, signifying a reduction of 36.41% between LP and HPS (Figure 1d). Soluble sugar is an important reservoir of energy for almost every single living creature on this planet. Plant species produce this organic substance through several important processes like photosynthesis and during respiratory breakdown. During the present study a significant decline (*P* < 0.05) in soluble sugar content was observed in *P. dulce* under fly ash stress. Soluble sugars concentration governs the sensitivity of plants to FA stress and also point to the physiological activity of a plant. A decline in total soluble sugar content indicates the intervention of light absorption triggered by FA dust deposition over the surface of leaves. Similar reports of decreased sugar content of crops, chlorophyll degradation and decreased CO_2_ fixation, and increased respiration, due to cement dust was also reported by Tripathi and Gautam [67].

**Figure 2:**
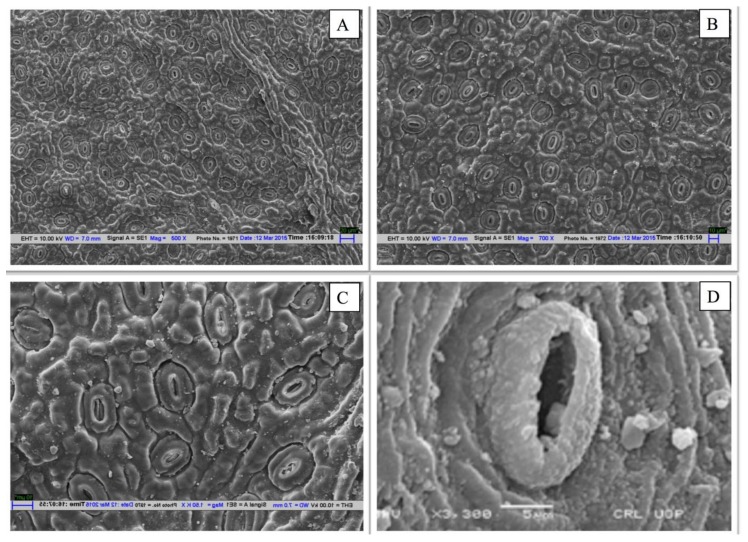
Leaf stomatal morphology of *P. dulce* as seen under SEM. (**A**&**B**) collection from LPS showing normal stomatal morphology. (**C**&**D**) collection from HPS showing stomata in deteriorated conditions with FA dust accumulation.

Plant foliar sulphur content increased significantly (*F* = 64.46, *P* < 0.01) from LPS to HPS. The maximum sulphur content of 255.40 µ molg^−1^ was recorded at HPS, followed by MPS (223.30 µ molg^−1^), while the minimum (204.59 µ molg^−1^) was recorded at LPS, resulting in a difference of 53.96% between the LPS and HPS (Figure 1d). In plants leaf sulphur content at several developmental stages, is determined by the atmospheric as well as soil sulphur uptake by the plant. It has been demonstrated that plants exposed to SO_2_ accumulate sulphur mainly through open stomata on leaves present on aerial plant parts [53]. Constant upsurge in sulphur content in leaves of *P. dulce* indicates the magnitude of pollution load at the sites of highest pollution. This also points toward the letdown of the detoxification mechanism involved in the elimination of the surplus S-derived bisulphite and sulphite ions. A higher amount of sulphur beyond permissible limits has potential damaging effects on plants, chiefly under synergetic effects of other contaminants [14,52]. A significant increase in intercellular carbon dioxide (*F* = 14.78, *P* < 0.01) and stomatal index (*F* = 68.83, *P* < 0.01) was observed with an increase in fly ash stress. Intercellular CO_2_ concentration enhanced from LPS to site HPS, displaying a maximum enhancement of 12.95%. However, at MPS, an increase of 5.77% was observed when compared to LPS. Stomatal conductance shows a significant (*F* = 5.79, *P* < 0.001) decline of 46.95% between the control (LPS) and the highly polluted site. A significant decrease was also observed in the case of NPR (*F* = 9.10, *P* < 0.01) under higher levels of FA contamination in the environment. In *P. dulce*, net photosynthetic rate (P_N_) showed a reduction of 16.60% and 37.31% between MPS and HPS. Stomatal index (SI) shows an augmentation of 10.69% from LP to HPS, as shown in Table 1. In response to pollution, Stomatal index may either increase or decrease as an avoidance strategy or an adaptive trait. Stomatal appearance was indubitably affected by FA stress as manifested from the SEM images of epidermal surfaces (Figure 2).

A decrease in the net photosynthetic rate led to elevated levels of intercellular carbon dioxide. A reduction in photosynthetic rate and stomatal conductance in tree species has been ascribed to dust load, as stated by Chaturvedi, et al. [25]. Any decrease in stomatal conductance under FA contamination may be ascribed to pore size reduction, high intercellular carbon dioxide concentration and lowered photosynthetic rate due to FA dust deposition (Figure 2). The noticeable decline in the stomatal index at the contaminated sites was similar to the findings with poplar clones, wherein SI condensed under raised CO_2_ level in the growing leaves [58].

**Figure 3:**
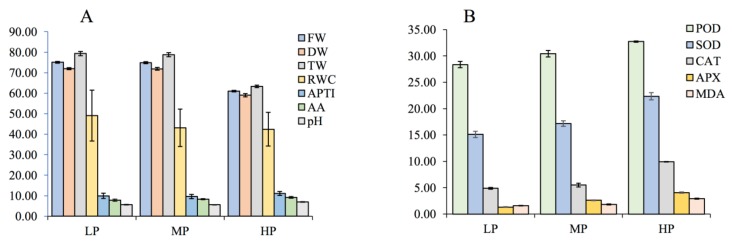
Site-specific variation in APTI of *P. dulce* (**A**) APTI and related parameters (**B**) Variation in the antioxidant enzymes activities of POD, SOD, CAT and APX (EU mg^−1^ protein) at three different sites and MDA content (µg g^−1^ fw). Data represents mean ± SD (*n* = 5).

The Analysis of variance (ANOVA) results exhibited a significant (*P* < 0.001) decrease in various parameters such as pH, FW, DW and TW of *P. dulce* at three different sites, whereas AA, and RWC, and APTI showed an increasing trend with an increase in the FA stress. Among the four parameters of APTI, AA content (mg g^−1^ fresh wt.) and RWC were found to increase with FA stress (Figure 3a). Being an antioxidant, AA counters the impact of air pollution on vegetation. Air pollution tolerance in plants is attributed to AA. Occurrence of higher AA content in leaves under water stress conditions might be an approach for protecting membranes of the thylakoid from oxidative destruction [1]. Several investigators have reported analogous results on AA content of leaves [68,69]. Similarly, higher pH in plant leaf extracts may lead to pollution tolerance in plants [1]. Leaf relative water content is related to protoplasmic permeability; consequently, plants with higher RWC are conceivably more tolerant to air pollutants. Physiological balance under stressful environmental conditions is maintained by plants through higher values of leaf relative water content especially when rates of transpiration normally stay higher. RWC in plants is considered to be an indicator of drought resistance [70].

The MDA content increased significantly from LPS to HPS sites representing an increment of 84.81% between the control and the maximum pollution site, as shown in Figure 3b. In contrast to the above parameters at HPS site, a remarkable increase was observed in antioxidant enzyme activities in the leaves of *P. dulce*, as compared to LPS. The minimum SOD activity of 15.11 U mg^−1^ FW was perceived at LPS, which progressively increased to 17.15 U mg^−1^ FW (13.5%) at MPS and reached a maximum of 22.34 U mg^−1^ FW (47.85%) at HPS, while the activity of APX increased from 1.33 U mg^−1^ FW at LPS to 2.62 U mg^−1^ FW (97%) at MPS and 4.06 U mg^−1^ FW (207.58%) at HPS. Similarly, CAT activity also increased significantly from LPS to HPS, the minimum value of 4.89 µmol min^−1^ observed at LPS increased to 5.51 µmol min^−1^ (12.67%) at MPS and finally to 9.92 µmol min^−1^ (102.86%) at HPS. The POD activity enhanced from a minimum of 28.35 µmol min^−1^ at LPS to a maximum of 32.72 µmol min^−1^ at HPS showing an increment of 15.41% between LPS and HPS. ANOVA indicated significant (*P* < 0.001) increase between antioxidants at three sites, as shown in Table 2. The activity of antioxidant enzymes often seems to be hooked to levels and types of stress but certain other factors like ecological conditions, duration of stress exposure and the degree of tolerance of the plant species also play a pivotal role in determination the antioxidant activity [70,71]. Various authors have reported their assessments on diverse facets of antioxidant enzyme playing their pivotal role in the biosynthesis of several important molecules, in the transport system and above all stress tolerance. An increase in the antioxidant enzymes like SOD, CAT, POD, and GR in roots, leaves and shoots of chickpea grown on fly ash, with increasing FA concentration in soil was reported by Pandey, et al. [17].

Transcriptional levels of several antioxidative enzymes, SOD, CAT, APX and POD in fly ash contaminated *P. dulce* plants was evaluated by qRT-PCR. Alleviated transcriptional expression profiles were observed in the case of SOD, POD, CAT and APX genes with an increase in pollution levels. Increased transcriptional levels of the genes in MPS and HPS plants were matched with those of growing at LPS levels. SOD, POD, CAT, and APX genes showed almost four-fold, increased gene expression level at MPS, while a 6 to 7-fold increase in the level of expression was observed at HPS (Figure 3b). Expression profiles of some stress related genes, viz. DREB and LEA were also studied. Compared to the LPS, increased expression levels were observed in *P. dulce* plants exposed to FA stress at both MPS and HPS. Since the genes have been observed to be induced under stressful conditions, the transcriptional levels were observed to be 3 to 5-fold in the case of LEA and 4–5-fold in case of DREB **(**Figure 3b,c).

Various significant metabolic and physiological pathways operating in plants are the major sources of ROS. However, ROS accumulation under stressful environments could reassure cell disintegration, membrane damage, membrane lipid peroxidation, and can even lead to cell death [72,73]. With the passage of time plants have developed an extensive redox balancing mechanism based on the enzymatic and nonenzymatic antioxidant systems [74]. SOD being the first line of defense in the multifaceted enzymatic antioxidant defense systems, plays a significant role in converting O_2_* to O_2_ and H_2_O_2_. Afterwards H_2_O_2_ is broken down by POD, and CAT to H_2_O [70,75,76]. Several environmental perturbations in plants lead to higher antioxidant enzyme levels [72] correspondingly a substantial surge in CAT, POD, and SOD, activities were perceived in *P. dulce* plants after exposure to fly ash stress (Figure 3b).

**Figure 4:**
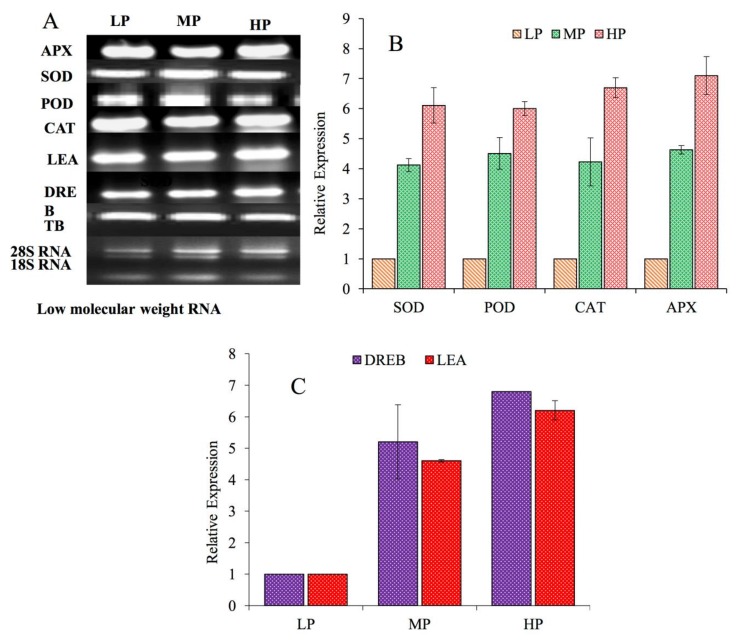
Transcriptional expression of antioxidant and stress related genes in *P. dulce* under fly ash stress. (**A**) Semi quantitative PCR showing expression. (**B**&**C**) Relative gene expression of antioxidant and stress related genes through Real time PCR

Transcriptional expression levels were also detected in the case of an antioxidant defense system leading to stress tolerance in *P. dulce* plants. During the present study increased transcript expression of the CAT gene was observed under fly ash stress (Figure 4A,B). Similar reports of increased CAT expression were reported in the case of maize and *Hordeum vulgare* seedling under drought stress conditions [77]. Expression of antioxidant related genes is reported to be upregulated under abiotic stress conditions [78]. With several remarkable functions, LEA proteins are recounted to exist in diverse organisms that shield proteins from clumping due to desiccation or osmotic stresses prompted by different environmental conditions [79].

With respect to fly ash stress profuse transcript profiles of the LEA gene were perceived in *P. dulce* plants (Figure 4A,C). DREBs belong to an essential class of transcriptional factors that regulate several downstream stress responsive genes involved in stress tolerance. DREB transcriptional factors, such as DREB2 and DREB1 expression in several plants have been observed to be induced through dehydration and cold activated signalling pathways [80].

From earlier experiments it was publicized that the expression of *AtDREBI* occurs in response to cold stress but not due to drought and salinity stress [81]. Correspondingly, transcriptional expression of DREB2A and DREB2B was observed to be induced under high salt and osmotic stress rather than cold stress [81]. Abiotic stresses like salt, cold and drought induce transcriptional expression of PgDREB2A gene in *Pennisetum glaucum* [82]. Analogous expression profiles were detected in the case of the DREB gene expressed in *P. dulce* under fly ash stress, thus demonstrating its potential role in stress tolerance (Figure 4C).

## 4. Conclusions

Significant changes take place in various foliar, morphological, biochemical parameters, leaf attributes and antioxidant enzymes of *P. dulce* grown at different sites around a dumping site of Badarpur thermal power plant of the NCR of Delhi. However, despite all of these changes the APTI value of the plant species at MPS and HPS was greater, as compared to LPS, and the plants were flourishing very well at the polluted sites. It may, therefore, be concluded that *P. dulce* is a resistant plant species resistant to FA pollution. Hence, this species may be used for remediation of FA dumps and also used as the biomarker of /and for mitigation of FA pollution around thermal power plants. Further, plantation of this tree species would help in greening urban environments and for pollution control.

## Figures and Tables

**Table 1:** Functional stomatal characteristics and Leaf attributes of *P. dulce* at three sites.

Sites	Intercellular Carbon Dioxide (mmol, CO_2_ m^−2^ S^−1^)	Stomatal Conductance (mmol, CO_2_ m^−2^ S^−1^)	P_N_(mmol m^−2^ S^−1^)	Stomatal Index (%)	SLA (cm^2^)	Leaf Length (cm)	Leaf Width (cm)
**LPS**	313.22 ± 24.70	1.15 ± 0.78	5.36 ± 1.62	14.12 ± 0.48	6.31 ± 0.85	9.16 ± 0.61	3.56 ± 0.26
**MPS**	331.38 ± 25.93	0.87 ± 0.52	4.47 ± 1.60	14.15 ± 0.43	4.23 ± 0.21	6.93 ± 1.20	2.59 ± 0.25
**HPS**	353.80 ± 26.11	0.61 ± 0.24	3.36 ± 1.76	15.63 ± 0.63	3.06 ± 0.23	4.94 ± 0.90	1.21 ± 0.27

SLA = Specific Leaf Area; P_N_ = Net photosynthetic rate.

**Table 2:** ANOVA Summary of APTI related parameters and antioxidant enzymes at three experimental sites of *P. dulce.*

Parameters	Sum of Squares	Df	Mean Square	F	Sig.
Ascorbic acid	23.32	74	11.66	54.76	***
pH	32.35	74	16.18	441.83	***
FW	3268.12	74	1634.06	8006.07	***
DW	2768.57	74	1384.28	2976.34	***
TW	4135.29	74	2067.65	2685.09	***
RWC	71.17	74	35.58	0.27	NS
APTI	63.11	74	31.55	21.97	***
SOD	694.25	74	347.13	961.79	***
APX	93.39	74	46.70	6467.29	***
CAT	377.16	74	188.58	3644.43	***
POD	238.34	74	119.17	488.64	***
MDA	25.20	74	12.60	2282.15	***

*** *P* < 0.001, ** *P* < 0.05, NS, Non-significant.

**Table 3:** Correlation coefficients for linear regression between biochemical parameters, leaf attributes of *P. dulce* at three different sites.

	Sites	Chl. a	Chl. b	CTDS	T. Chl.	NPR	NRA	Nitrate	Nitrogen	Proline	SPT	RS	SCc	SI	SC	ICD	SPM	SLA	LL	LW	AA	pH	FW	DW	TW	RWC	APTI	SOD	APX	CAT	POD	MDA
Sites	1																															
Chl. a	−0.649 ^a^	1																														
Chl. b	−0.551 ^a^	0.854 ^a^	1																													
CTDS	−0.565 ^a^	0.828 ^a^	0.893 ^a^	1																												
T. Chl.	−0.630 ^a^	0.973 ^a^	0.951 ^a^	0.889 ^a^																												
NPR	−0.448 ^a^	0.644 ^a^	0.634 ^a^	0.512 ^a^	0.664 ^a^	1																										
NRA	−0.314 ^a^	0.624 ^a^	0.295 ^b^	0.357 ^a^	0.502 ^a^	0.608 ^a^	1																									
Nitrate	−0.686 ^a^	0.508 ^a^	0.247 ^b^	0.176	0.411 ^a^	0.585 ^a^	0.699 ^a^	1																								
Nitrogen	0.718 ^a^	−0.619 ^a^	−0.397 ^a^	−0.482 ^a^	−0.544 ^a^	−0.357 ^a^	−0.619 ^a^	−0.672 ^a^	1																							
Proline	0.974 ^a^	−0.623 ^a^	−0.516 ^a^	−0.535 ^a^	−0.599 ^a^	−0.381 ^a^	−0.291 ^b^	−0.634 ^a^	0.686 ^a^	1																						
SPT	−0.236 ^b^	0.672 ^a^	0.535 ^a^	0.503 ^a^	0.636 ^a^	0.740 ^a^	0.768 ^a^	0.496 ^a^	−0.344 ^a^	−0.192	1																					
RS	−0.313 ^a^	0.318 ^a^	0.520 ^a^	0.379 ^a^	0.420 ^a^	0.660 ^a^	0.058	0.161	0.165	−0.268 ^b^	0.348 ^a^	1																				
SCc.	0.792 ^a^	−0.496 ^a^	−0.578 ^a^	−0.552 ^a^	−0.551 ^a^	−0.334 ^a^	0.093	−0.248 ^b^	0.331 ^a^	0.799 ^a^	−0.077	−0.439 ^a^	1																			
SI	0.711 ^a^	−0.720 ^a^	−0.477 ^a^	−0.543 ^a^	−0.639 ^a^	−0.410 ^a^	−0.510 ^a^	−0.527 ^a^	0.725 ^a^	0.725 ^a^	−0.323 ^a^	−0.067	0.493 ^a^	1																		
SC	−0.372 ^a^	0.737 ^a^	0.681 ^a^	0.865 ^a^	0.740 ^a^	0.500 ^a^	0.557 ^a^	0.188	−0.406 ^a^	−0.351 ^a^	0.595 ^a^	0.278 ^b^	−0.292 ^b^	−0.427 ^a^	1																	
ICD	0.551 ^a^	−0.236 ^b^	−0.290 ^b^	0.004	−0.269 ^b^	−0.472 ^a^	−0.046	−0.609 ^a^	0.273 ^b^	0.494 ^a^	−0.114	−0.380 ^a^	0.404 ^a^	0.250 ^b^	0.227 ^b^	1																
SPM	0.408 ^a^	−0.001	0.107	0.037	0.047	−0.144	0.022	−0.227	0.414 ^a^	0.397 ^a^	0.190	0.111	0.418 ^a^	0.391 ^a^	0.146	0.182	1															
SLA	−0.922 ^a^	0.566 ^a^	0.414 ^a^	0.432 ^a^	0.520 ^a^	0.432 ^a^	0.384 ^a^	0.769 ^a^	−0.701 ^a^	−0.889 ^a^	0.215	0.171	−0.618 ^a^	−0.621 ^a^	0.309 ^a^	−0.519 ^a^	−0.406 ^a^	1														
LL	−0.884 ^a^	0.555 ^a^	0.452 ^a^	0.463 ^a^	0.530 ^a^	0.342 ^a^	0.230 ^b^	0.576 ^a^	−0.617 ^a^	−0.845 ^a^	0.115	0.339 ^a^	−0.688 ^a^	−0.603 ^a^	0.316 ^a^	−0.445 ^a^	−0.368 ^a^	0.791 ^a^	1													
LW	−0.961 ^a^	0.677 ^a^	0.593 ^a^	0.585 ^a^	0.666 ^a^	0.467 ^a^	0.300 ^a^	0.645 ^a^	−0.654 ^a^	−0.938 ^a^	0.294 ^b^	0.358 ^a^	−0.792 ^a^	−0.693 ^a^	0.378 ^a^	−0.566 ^a^	−0.332 ^a^	0.854 ^a^	0.822 ^a^	1												
AA	0.768 ^a^	−0.490 ^a^	−0.334 ^a^	−0.363 ^a^	−0.439 ^a^	−0.446 ^a^	−0.452 ^a^	−0.690 ^a^	0.619 ^a^	0.735 ^a^	−0.316 ^a^	−0.217	0.453 ^a^	0.621 ^a^	−0.275 ^b^	0.494 ^a^	0.244 ^b^	−0.782 ^a^	−0.568 ^a^	−0.739 ^a^	1											
pH	0.834 ^a^	−0.679 ^a^	−0.586 ^a^	−0.546 ^a^	−0.664 ^a^	−0.481 ^a^	−0.248 ^b^	−0.506 ^a^	0.667 ^a^	0.817 ^a^	−0.213	−0.231 ^b^	0.684 ^a^	0.802 ^a^	−0.304 ^a^	0.550 ^a^	0.506 ^a^	−0.746 ^a^	−0.714 ^a^	−0.835 ^a^	0.692 ^a^	1										
FW	−0.871 ^a^	0.687 ^a^	0.600 ^a^	0.581 ^a^	0.674 ^a^	0.411 ^a^	0.215	0.470 ^a^	−0.618 ^a^	−0.868 ^a^	0.215	0.310 ^a^	−0.752 ^a^	−0.808 ^a^	0.329 ^a^	−0.506 ^a^	−0.391 ^a^	0.727 ^a^	0.764 ^a^	0.885 ^a^	−0.711 ^a^	−0.961 ^a^	1									
DW	−0.861 ^a^	0.673 ^a^	0.576 ^a^	0.566 ^a^	0.656 ^a^	0.400 ^a^	0.220	0.465 ^a^	−0.619 ^a^	−0.866 ^a^	0.217	0.283 ^b^	−0.748 ^a^	−0.821 ^a^	0.324 ^a^	−0.491 ^a^	−0.401 ^a^	0.722 ^a^	0.743 ^a^	0.876 ^a^	−0.714 ^a^	−0.954 ^a^	0.993 ^a^	1								
TW	−0.874 ^a^	0.700 ^a^	0.600 ^a^	0.586 ^a^	0.682 ^a^	0.432 ^a^	0.251 ^b^	0.492 ^a^	−0.626 ^a^	−0.871 ^a^	0.236 ^b^	0.310 ^a^	−0.740 ^a^	−0.821 ^a^	0.347 ^a^	−0.495 ^a^	−0.394 ^a^	0.744 ^a^	0.761 ^a^	0.885 ^a^	−0.728 ^a^	−0.961 ^a^	0.994 ^a^	0.990 ^a^	1							
RWC	0.084	−0.135	−0.044	−0.062	−0.099	−0.095	−0.177	−0.071	0.158	0.151	−0.064	0.008	0.099	0.309 ^a^	−0.065	−0.074	0.122	−0.051	−0.104	−0.073	0.053	0.071	−0.070	−0.118	−0.103	1						
APTI	0.574 ^a^	−0.423 ^a^	−0.270 ^b^	−0.284 ^b^	−0.371 ^a^	−0.337 ^a^	−0.324 ^a^	−0.432 ^a^	0.529 ^a^	0.611 ^a^	−0.163	−0.111	0.433 ^a^	0.688 ^a^	−0.177	0.296 ^b^	0.382 ^a^	−0.528 ^a^	−0.488 ^a^	−0.553 ^a^	0.593 ^a^	0.604 ^a^	−0.594 ^a^	−0.631 ^a^	−0.622 ^a^	0.796 ^a^	1					
SOD	0.952 ^a^	−0.620 ^a^	−0.517 ^a^	−0.533 ^a^	−0.598 ^a^	−0.329 ^a^	−0.209	−0.556 ^a^	0.660 ^a^	0.956 ^a^	−0.166	−0.250 ^b^	0.800 ^a^	0.745 ^a^	−0.306 ^a^	0.494 ^a^	0.413 ^a^	−0.840 ^a^	−0.827 ^a^	−0.944 ^a^	0.749 ^a^	0.887 ^a^	−0.943 ^a^	−0.938 ^a^	−0.941 ^a^	0.053	0.569 ^a^	1				
APX	0.997 ^a^	−0.647 ^a^	−0.549 ^a^	−0.561 ^a^	−0.628 ^a^	−0.417 ^a^	−0.284 ^b^	−0.663 ^a^	0.694 ^a^	0.978 ^a^	−0.208	−0.306 ^a^	0.802 ^a^	0.712 ^a^	−0.365 ^a^	0.544 ^a^	0.390 ^a^	−0.917 ^a^	−0.883 ^a^	−0.961 ^a^	0.762 ^a^	0.842 ^a^	−0.884 ^a^	−0.875 ^a^	−0.887 ^a^	0.079	0.571 ^a^	0.962 ^a^	1			
CAT	0.913 ^a^	−0.669 ^a^	−0.568 ^a^	−0.566 ^a^	−0.650 ^a^	−0.425 ^a^	−0.251 ^b^	−0.534 ^a^	0.656 ^a^	0.908 ^a^	−0.202	−0.320 ^a^	0.746 ^a^	0.806 ^a^	−0.333 ^a^	0.519 ^a^	0.420 ^a^	−0.788 ^a^	−0.812 ^a^	−0.908 ^a^	0.750 ^a^	0.955 ^a^	−0.989 ^a^	−0.981 ^a^	−0.988 ^a^	0.089	0.620 ^a^	0.961 ^a^	0.923 ^a^	1		
POD	0.965 ^a^	−0.614 ^a^	−0.500 ^a^	−0.490 ^a^	−0.587 ^a^	−0.409 ^a^	−0.262 ^b^	−0.687 ^a^	0.627 ^a^	0.949 ^a^	−0.200	−0.291 ^b^	0.801 ^a^	0.699 ^a^	−0.285 ^b^	0.597 ^a^	0.397 ^a^	−0.881 ^a^	−0.816 ^a^	−0.951 ^a^	0.732 ^a^	0.812 ^a^	−0.853 ^a^	−0.848 ^a^	−0.854 ^a^	0.082	0.557 ^a^	0.931 ^a^	0.967 ^a^	0.884 ^a^	1	
MDA	0.932 ^a^	−0.663 ^a^	−0.578 ^a^	−0.595 ^a^	−0.650 ^a^	−0.385 ^a^	−0.215	−0.508 ^a^	0.648 ^a^	0.937 ^a^	−0.190	−0.285 ^b^	0.811 ^a^	0.790 ^a^	−0.352 ^a^	0.487 ^a^	0.416 ^a^	−0.808 ^a^	−0.793 ^a^	−0.931 ^a^	0.752 ^a^	0.931 ^a^	−0.976 ^a^	−0.973 ^a^	−0.972 ^a^	0.091	0.611 ^a^	0.979 ^a^	0.943 ^a^	0.983 ^a^	0.914 ^a^	1

a. Correlation is significant at the 0.01 level; b. Correlation is significant at the 0.05 level. *Chl. a,* Chlorophyll *a, Chl. b;* Chlorophyll *b; CTDS*, Carotenoids; *T. Chl*. Total Chlorophyll; *NPR,* Net photosynthetic rate; *SPT,* Soluble protein; *RS,* Reducing sugar, *SCc*. Sulphur content, *SI,* stomatal index, *SC*, Stomatal conductance, *ICD,* Intercellular Carbon dioxide concentration, *SPM,* Suspended Particulate Matter, *SLA,* Specific leaf area, *LL,* leaf length, *LW,* Leaf width, *AA,* Ascorbic acid, *FW,* Fresh weight, *DW,* Dry weight, *TW,* Turgid weight, *RWC,* Relative water content, *APTI,* Air pollution tolerance index, *SOD,* Superoxidase dismutase, *APX,* Ascorbate peroxidase, *POD,* Peroxidase, *MDA,* Malondialdehyde.
